# Comparing the effectiveness of stimulation using rhTSH and thyroid hormone withdrawal in the treatment of thyroid cancer

**DOI:** 10.1186/1756-6614-8-S1-A16

**Published:** 2015-06-22

**Authors:** Aldona Kowalska

**Affiliations:** 1 Endocrinology Department; Holycross Cancer Center, Kielce, Poland

## 

Thyroid cancer (TC) is the most common neoplasm of the endocrine system. In 2011, the standardized incidence rate in Poland was 7.4 for women and 1.7 for men [1]. This rate is characterized by a steady increase. However, it is not accompanied by an increase in mortality [2,3]. On the contrary, a significant reduction is observed due to diagnosing cancer at earlier stages and improved treatment effectiveness [4].

Over 90% of all TC are differentiated thyroid cancers (DTC), which are characterized by favorable prognosis, i.e. 10-year survival in 90-95% of cases [5]. As a result of treatment, more than 80% of patients recover, although in 15% of cases local recurrence is observed, with distant metastases being diagnosed in 5-10% of cases. Relapse frequently occurs within the first five years, but there have been reports of recurrences or distant metastases even 40 years later; therefore, lifetime oncological follow-up is required [6].

Taking into account the very good prognosis and the need for long-term monitoring, patients should be offered the safest and most comfortable procedures.

The biggest burden for patients with DTC resulting from oncological treatment and follow up is the use of a radioiodine (^131^I) and periods of hypothyroidism required to evaluate TSH-stimulated thyroglobulin (Tg) - a sensitive and specific marker for DTC.

The use of ^131^I is associated with a dose-dependent increase in the risk of secondary neoplasms: leukemia, bone cancer, stomach and colorectal cancer, salivary gland cancer and soft tissue tumors. Compared to the general population, in patients treated with ^131^I an overall 27% increase in the risk of other tumors was observed. The adverse effects of ^131^I are also manifested as impaired function of salivary glands, and parotid glands in particular [7,8].

Periods of hypothyroidism lasting for approximately 4-6 weeks are associated with disturbances in the patients' symptoms of hypothyroidism, deteriorating patients' physical, intellectual and social functioning. Additional treatment is often needed due to exacerbation of comorbid conditions and inability to work [9].

The introduction of recombinant human TSH (rhTSH) was a breakthrough in the care of patients with DTC.

Recombinant human TSH (rhTSH) is a protein produced by the ovarian cell lines of Chinese hamsters transfected with DNA that encodes both subunits of the protein. The bioactivity of recombinant TSH depends on the degree of saturation of the carbohydrate component with the sialic acid residues, and is as high as for endoTSH in overt hypothyroidism and significantly higher than for endoTSH in the state of hormonal balance. Recombinant TSH strongly stimulates iodine uptake as well as Tg and thyroid hormone synthesis in both thyrocytes and DTC cells. The results of studies evaluating the impact of rhTSH on ^131^I pharmacokinetics are of great importance, indicating a decrease in isotope radiotoxicity (reduction of exposure dose for the bone marrow by a third) by accelerating the renal clearance of iodine and reduction of the effective half-life of ^131^I in the whole body from 0.54 +/- 0.1 day to 0.43 +/- 0.1 day. At the same time, the effective half-life of ^131^I is extended in the thyroid gland residues, which is a beneficial effect that determines effectiveness of the treatment [10-12].

Registered indications relate to the use of rhTSH for ablation in patients after total thyroidectomy with no evidence of distant metastases, as well as in monitoring the course of the disease. Numerous studies [13,14] confirm the equal effectiveness of ablation for ^131^I at 1100 MBq and 3700 MBq regardless of the method of stimulation: rhTSH or thyroid hormone withdrawal (HiLo -T1-T3, N0, N1, Mo; ESTIMABL -T1-T2, No, N1, M0).

The results of multicenter studies in patients with T4 carried out by Bartenstein et al. and published in 2013 also confirmed the high efficacy of ablation using rhTSH in the higher risk group [15].

The results of a ten-year follow-up study in patients undergoing ablation using rhTSH, presented by Malinoro et al., demonstrated equal frequency of recurrence episodes, distant metastases and persistent disease compared with patients treated with thyroid hormone withdrawal [16].

Recombinant human TSH has not been registered for the treatment of patients with metastatic diseases; however, such attempts are being made. The first reports of a successful treatment with ^131^I after rhTSH stimulation in patients with distant metastases were published in 2000 by Luster [17] et colleagues, and in 2003 by Jarząb et al. [18]. Studies conducted by Tal et al. demonstrated equal 5-year survival rates in patients with DTC metastases to the lungs and bones, regardless of the method of preparation for the ^131^I therapy: rhTSH or thyroid hormone withdrawal [19].

An important indication for the use of rhTSH is disease monitoring. The first assessment concerning the effectiveness of ablation therapy is carried out 6-12 months after radioiodine treatment. The assessment includes rhTSH-stimulated Tg levels, anti-Tg antibodies levels, thyroid ultrasound and whole-body scintigraphy. In more than 80% of patients, serum Tg levels reach the highest values at day 5 after administration of rhTSH. Evaluation of serum Tg levels after TSH stimulation is the most effective method of disease monitoring. The sensitivity of Tg measurement during the treatment with L-T4 is definitely lower: in 20% of patients with metastases to the lymph nodes and in 5% with distant metastases it may be false negative [20].

Periodic monitoring of patients with complete remission of the disease must be carried out over a period of many years. In patients with a low risk of relapse, in the absence of anti-Tg, it is possible to omit the whole-body scintigraphy and only to evaluate the stimulated Tg and perform the ultrasound examination of the neck and chest X-ray [20].

The use of rhTSH in preparing patients for the treatment of metastatic disease is justified if the patient's condition does not allow a break in LT4 administration in fear of exacerbation of the symptoms of neoplasm or comorbidities, and if it is impossible to achieve endogenous stimulation due to hypopituitarism or hormonally active metastases.

New opportunities for the implementation of rhTSH include: improvement of the efficiency of imaging patients using FDG PET/CT after rhTSH administration, diagnosis of congenital hypothyroidism, treatment of nodular goiter with ^131^I, assessment of thyroid reserve in elderly patients, testing of TSH-dependent immune system genes, evaluation of differences in the metabolism of adipose tissue and secretion of adipokines [21].

## Summary

• Diagnosis and treatment with ^131^I is equally effective regardless of the method of TSH stimulation: with rhTSH or with LT4 withdrawal.

• Recombinant human TSH extends the effective half-life of radioiodine in the thyroid residues, which may increase the effectiveness of the treatment.

• Minimized radiotoxicity of ^131^I may reduce the risk of secondary malignancies.

• Assistance is required in avoiding hypothyroidism and related symptoms, deterioration of quality of life and inability to work professionally.

• Despite the high price of rhTSH, the total economic analysis of the cost/benefit ratio suggests that its use is favorable.
